# Training under pressure mirrors competition: technical-tactical insights from high-level male padel players

**DOI:** 10.3389/fspor.2025.1505817

**Published:** 2025-02-11

**Authors:** Rafael Conde-Ripoll, Adrián Escudero-Tena, Vicente Javier Clemente-Suárez, Eduardo Navarro Jimenez, Álvaro Bustamante-Sánchez

**Affiliations:** ^1^ Universidad Europea de Madrid, Department of Sports Sciences, Faculty of Medicine, Health and Sports, Madrid, Spain; ^2^ Research Group on Culture, Education and Society, Universidad de la Costa, Barranquilla, Colombia; ^3^ Faculty of Health Sciences, Universidad Simón Bolívar, Barranquilla, Colombia

**Keywords:** racket sport, performance, game analysis, notational analysis, pressure training

## Abstract

**Introduction:**

This study aimed to analyse the differences between pressure training and official competition matches in high-level male padel players from Finland, focusing on the proportion of points won by serving and returning players, the frequency of break points and golden points, and the effectiveness of the last shot.

**Methods:**

A total of 4,417 points from 38 matches played during the 2022 and 2023 seasons were analyzed.

**Results:**

The results revealed no significant association between match type (pressure training vs. competition) and the distribution of winners, forced errors, or unforced errors (*p* = 0.867). Similarly, the frequency of break points, non-break points, golden points, and non-golden points remained consistent across both match types, regardless of the service situation (serving or returning players). While a relationship was observed between shot type and match type in forced errors (*p* = 0.024), the overall shot effectiveness remained comparable across different shot types.

**Discussion:**

In conclusion, this study suggests that high-level male padel players display comparable technical-tactical performance in pressure training and official competition matches.

## Introduction

From repetitive shot drills fed by coaches from a basket to more open situations such as training matches, padel players at all levels showcase their training routines on social media. Ideally, the training process in sports should involve a holistic approach, optimizing athletes' physical, technical, cognitive, and psychological capacities (1). The principle “practice how you play” (2) highlights the importance of mirroring competitive conditions during training, as previous research suggests that closer alignment between training and competition enhances skill and behavioral transfer (3–5).

In padel, players face constant decision-making (6) and pressure-filled situations, such as break points (e.g., 15/40 or 30/40) or golden points (40/40 under no-advantage rules). Pressure, defined as “the presence of situational motivators for achieving optimal, maximal, or superior performance” (7), is perceived differently across athletes due to factors like personal goals or external rewards (8–10). Although physical demands during matches remain consistent (11), athletes’ cognitive appraisal and emotional regulation under pressure often dictate their performance outcomes (12, 13). Poor pressure management of these demands can lead to performance deterioration (14, 15), while effective coping strategies are associated with improved outcomes (16–18). As a result, coaches are encouraged to implement ecological training practices, which simulate match scenarios by increasing task demands or the consequences of errors (19, 20).

Technical-tactical performance analysis also plays a critical role in training and competitions, providing valuable insights into player actions and strategies (21). A key metric in padel is the effectiveness of the last shot, classified as a winner, a forced error, or an unforced error (22). In men's professional padel, winners often result from well-executed smashes (23–25), while forced and unforced errors frequently involve forehand and backhand volleys, highlighting key areas for improvement (24).

Despite the importance of pressure training and technical-tactical analysis, research on these aspects in high-level padel remains limited. This study aims to fill this gap by comparing pressure training and competitive match scenarios among high-level male players in Finland. Specifically, we analyzed differences in points won by servers and returners, the occurrence of break points and golden points, and the effectiveness of the last shot. We hypothesized that pressure training would closely replicate competition conditions, offering a reliable framework for enhancing performance.

## Method

### Research design

This research follows an empirical methodology, specifically employing a descriptive strategy. It is primarily observational in nature, being nomothetic, cross-sectional, and multidimensional (26). Although the study includes a form of intervention through pressure training, it remains observational as the pressure training simply simulated a high-stakes environment without controlling or manipulating player behavior. This approach allows for the observation of natural performance under varying conditions while maintaining the overall observational design.

### Sample

A total of 4,417 points were analyzed from 38 matches, including 18 pressure training matches and 20 official competition matches. Only points played in regular games were included; points from tiebreaks and super tiebreaks were excluded. The matches took place in Finland during 2022 and 2023. All competition matches were part of tournaments in the highest category, with points contributing to the Finnish Padel Federation ranking.

Participants were recruited using a convenience sampling approach, selecting players who were actively competing at the highest level in Finland. The sample comprised 30 male players, all ranked within the top 60 of the Finnish Padel Federation at the time of data collection. In the pressure training matches, 12 players from the top 25 participated. In the official competition matches, these 12 players were joined by an additional 18 players, all ranked within the top 60. Although not all players participated in both training and competition scenarios, this ranking distribution ensures that the level of play was consistently high across all analyzed matches.

Players were excluded if they were injured or unable to participate in either the training or competition matches during the study period. No additional exclusion criteria were applied.

The sample size was not determined using an *a priori* power calculation due to the recruitment approach. Instead, the study included all available data from matches and training sessions during the study period, providing a comprehensive and robust dataset for analysis.

All procedures were conducted according with ethical standards in sport and exercise science research (27) and in accordance with the Helsinki Declaration. The research was previously approved by the Ethics Committee for Research of the European University of Madrid with the code CIPI/22.303.

### Study variables

The following variables were defined and analyzed based on their categorical core and degree of openness (28):

- Match type: a difference was made between pressure training matches and official competition matches.
- Break point type: a difference was made between break points and non-break points.
- Golden point type: a difference was made between golden points and non-golden points.
- Last shot serving situation: a difference was made between points in which the last shot was executed by the serving players and by the returning players.
- Serving efficiency: a difference was made between points won by the serving players and points won by the returning players.
- Effectiveness of the last shot: winner, forced error or unforced error. These categories are defined based on previous studies (29).
- Last shot type: a difference was made among bandeja, smash, fake smash, recovery smash, forehand volley, backhand volley, forehand bajada, backhand bajada, forehand, backhand, back wall forehand, back wall backhand, side wall forehand, side wall backhand, double wall forehand, double wall backhand, first serve, second serve, return, contrapared and other (cadete, willy..). The definition of each of these categories was based on previous studies (23, 30).

We acknowledge that this study did not include direct assessments of psychological factors such as mental fatigue, sleep quality, or mental strength, which are known to influence performance outcomes in high-pressure scenarios. The absence of these measures limits the ability to comprehensively evaluate the interplay between psychological and technical-tactical performance parameters.

### Procedure

The players were informed by the coach that they would undergo pressure training. Pressure training refers to an intervention designed to assist athletes in performing under pressure by deliberately exposing them to stressors during training sessions (19, 31, 32). In our study, players were recorded during the practice matches while their technical-tactical performance was evaluated by the head coach of the first ever professional padel team of Finland.

An observer, a PhD student in Sports Sciences, certified padel coach and with a large number of published scientific research related to the topic of study, observed the matches live and recorded the study variables through an ad-hoc instrument. At the end of the collection process, an intra-observer reliability analysis was performed to ensure the veracity of the data collected. The observer reanalyzed a random sample of 6 matches (matches were recorded) to ensure enough relevant data to represent 10%–20% of the study sample (33). The mean intra-observer reliability was 0.90, considered almost perfect (34). In addition, another observer, a PhD in Sports Sciences, certified padel coach and with a large number of published scientific research related to the topic of study, also analyzed a random sample of 6 matches to calculate the average inter-observer reliability, which was 0.84 (34).

### Statistical analysis

An inferential analysis was performed to develop contingency tables, including the Chi-square (χ^2^) statistical test to obtain the association between variables. The strength of association between variables was also calculated, for which Cramer's V coefficient (Vc) was used (35). Crewson differentiates the strength of association according to the value, considering a small (<0.100), low (0.100–0.299), moderate (0.300–0.499) or high (>0.500) association. In addition, subsequent *Z*-tests were performed to compare column proportions, adjusting for *p*-values <0.05 according to Bonferroni (36). Contingency tables allowed identification of associations between variable categories through corrected standard residuals (CSR). Residuals >|1.96| betrayed cells with more or fewer cases than there should be (35). The significance level was set at *p* < 0.05 and statistical analysis was performed using the IBM SPSS (IBM SPSS Statistics for Windows, Version 27.0. IBM, Armonk, NY, USA) statistical package for Windows.

## Results

Table 1 shows the differences between pressure training and competition according to the effectiveness of the last shot. There is not any relationship between the effectiveness of the last shot and the match type (χ^2^ = 0.286; df = 2; *p* = 0.867; Vc = 0.008). In both practice and competitive matches, points predominantly concluded with unforced errors, followed by winners, and then forced errors.

**Table 1:** Differences between pressure training and competition according to the effectiveness of the last shot.

	Pressure training			Competition		
	*n*	%	CSR	*n*	%	CSR
Winners	834	37.3	0.2	847	37.0	−0.2
Forced errors	520	23.3	0.4	522	22.8	−0.4
Unforced errors	880	39.4	−0.5	918	40.1	0.5

*n*, number; %: percentage; CSR, corrected standard residuals.

Table 2 shows the differences between pressure training and competition according to the effectiveness of the last shot and last shot serving situation and to the serving efficiency. There is no significant relationship between the match type and the effectiveness of the last shot in points won by serving players (χ^2^ = 1.027; df = 2; *p* = 0.598; Vc = 0.021), nor by returning players (χ^2^ = 2.161; df = 2; *p* = 0.340; Vc = 0.031). Furthermore, no significant relationship is found between the pair that wins the points depending on the serving situation, and the match type (χ^2^ = 0.033; df = 1; *p* = 0.857; Vc = 0.003).

**Table 2:** Differences between pressure training and competition according (a) to the effectiveness of the last shot and last shot serving situation and (b) to the serving efficiency.

	Serving players						Returning players					
	Pressure training			Competition			Pressure training			Competition		
	*n*	%	CSR	*n*	%	CSR	*n*	%	CSR	*n*	%	CSR
Winners	544	47.8	0.6	539	47.2	−0.6	290	26.5	−0.2	308	26.9	0.2
Forced errors	168	14.7	−1.0	186	16.3	1.0	352	32.1	1.4	336	29.4	−1.4
Unforced errors	427	37.5	0.5	418	36.5	−0.5	453	41.4	−1.1	500	43.7	1.1
Total points won	1,349	60.4	0.2	1,375	60.1	−0.2	885	39.6	−0.2	912	39.9	0.2

*n*, number; %, percentage; CSR, corrected standard residuals.

Table 3 shows the differences between pressure training and competition according to the serving efficacy, break point type and golden point type. There is no significant relationship between the type of point (break point) and the match type (χ^2^ = 0.004; df = 1; *p* = 0.947; Vc = 0.001). There is no significant relationship between the break and non-break points won by the serving players, and the match type (χ^2^ = 0.220; df = 1; *p* = 0.639; Vc = 0.009). There is no significant relationship between the break and non-break points won by the returning players, and the match type (χ^2^ = 0.204; df = 1; *p* = 0.651; Vc = 0.011). There is no significant relationship between the type of point (golden point) and the match type (χ^2^ = 0.246; df = 1; *p* = 0.620; Vc = 0.007). There is no significant relationship between the golden and non-golden points won by the serving players, and the match type (χ^2^ = 2.515; df = 1; *p* = 0.113; Vc = 0.031). There is no significant relationship between the golden and non-golden points won by the returning players, and the match type (χ^2^ = 1.552; df = 1; *p* = 0.213; Vc = 0.030).

**Table 3:** Differences between pressure training and competition according to the serving efficacy, break point type and golden point type.

		Pressure training			Competition		
		*n*	%	CSR	*n*	%	CSR
Break points frequency	Break points	267	12.3	0.1	276	12.3	−0.1
	Non-break points	1,899	87.7	−0.1	1,975	87.7	0.1
Break and non-break points won by the servers	Break points won by the serving players	159	12.2	0.5	157	11.6	−0.5
	Non-break points won by the serving players	1,145	87.8	−0.5	1,196	88.4	0.5
Break and non-break points won by the returners	Break points won by the returning players	108	12.5	−0.5	119	13.3	0.5
	Non-break points won by the returning players	754	87.5	0.5	779	86.7	−0.5
Golden points frequency	Golden points	101	4.7	0.5	98	4.4	−0.5
	Non-golden points	2,065	95.3	−0.5	2,153	95.6	0.5
Golden and non-golden points won by the servers	Golden points won by the serving players	70	5.4	1.6	55	4.1	−1.6
	Non-golden points won by the serving players	1,234	94.6	−1.6	1,298	95.9	1.6
Golden and non-golden points won by the returners	Golden points won by the returning players	31	3.6	−1.2	43	4.8	1.2
	Non-golden points won by the returning players	831	96.4	1.2	855	95.2	−1.2

*n*, number; %, percentage; CSR, corrected standard residuals.

Table 4 shows the differences in winners, forced errors and unforced errors between pressure training matches and competitive matches. There is no significant relationship between the shot type and the match type neither in winners (χ^2^ = 16.349; df = 18; *p* = 568; Vc = 0.099) nor in unforced errors (χ^2^ = 24.765; df = 18; *p* = 0.132; Vc = 0.117). Nevertheless, there is a relationship between the shot type and the match type in forced errors (χ^2^ = 27.583; df = 15; *p* = 0.024; Vc = 0.163).

**Table 4:** Differences in winners, forced errors and unforced errors between pressure training matches and competitive matches.

Shot family		Winners				Forced errors				Unforced errors			
		P		C		P		C		P		C	
	Shot type	%	CSR	%	CSR	%	CSR	%	CSR	%	CSR	%	CSR
Without bounce	Smash	45.0^a^	1.3	41.9^a^	−1.3					4.1^a^	0.2	3.9^a^	−0.2
	Bandeja	11.6^a^	0.2	11.3^a^	−0.3	0.4^a^	0.6	0.2^a^	−0.6	14.8^a^	**2** **.** **3**	11.1^b^	−2.3
	Forehand volley	13.3^a^	−0.4	14.0^a^	0.4	8.5^a^	−1.2	10.7^a^	1.2	15.5^a^	−1.2	17.5^a^	1.2
	Backhand volley	11.0^a^	−0.6	11.9^a^	0.6	17.3^a^	0.2	16.9^a^	−0.2	15.5^a^	−1.2	17.5^a^	1.2
With bounce and without wall	Forehand	3.4^a^	−0.7	4.0^a^	0.7	10.8^a^	1.2	8.6^a^	−1.2	7.8^a^	−0.2	8.1^a^	0.2
	Backhand	1.8^a^	−1.4	2.8^a^	1.4	11.5^a^	−2.1	16.1^b^	**2** **.** **1**	8.6^a^	−0.7	9.6^a^	0.7
With bounce and with wall	Forehand bajada	3.5^a^	1.8	2.0^a^	−1.8	0.0^a^	−1.0	0.2^a^	1.0	2.8^a^	−1.3	3.9^a^	1.3
	Backhand bajada	0.7^a^	0.3	0.6^a^	−0.3					1.0^a^	−1.1	1.6^a^	1.1
	Back wall forehand	1.4^a^	−0.2	1.5^a^	0.2	7.7^a^	0.5	6.9^a^	−0.5	3.4^a^	−1.5	4.8^a^	1.5
	Back wall backhand	1.8^a^	1.3	1.1^a^	−1.3	7.1^a^	1.4	5.0^a^	−1.4	3.6^a^	0.7	3.1^a^	−0.7
	Out of the court	0.5^a^	−1.4	1.1^a^	1.4	1.5^a^	0.3	1.3^a^	−0.3	0.1^a^	1.0	0.0^a^	−1.0
	Side wall forehand	0.0^a^	−1.0	0.1^a^	1.0	4.0^a^	1.2	2.7^a^	−1.2	1.0^a^	1.2	0.5^a^	−1.2
	Side wall backhand	0.0^a^	−1.0	0.1^a^	1.0	3.3^a^	−2.4	6.5^b^	**2** **.** **4**	0.8^a^	1.7	0.2^a^	−1.7
	Double wall forehand	0.7^a^	0.0	0.7^a^	0.0	6.7^a^	−0.1	6.9^a^	0.1	0.5^a^	−1.1	0.9^a^	1.1
	Double wall backhand	0.4^a^	−1.2	0.8^a^	1.2	6.5^a^	−0.2	6.9^a^	0.2	1.8^a^	1.1	1.2^a^	−1.1
	Recovery smash	2.4^a^	−1.0	3.2^a^	1.0	0.8^a^	**2** **.** **0**	0.0^b^	−2.0	0.2^a^	1.4	0.0^a^	−1.4
Other	Contrapared	0.1^a^	1.0	0.0^a^	−1.0	7.7^a^	−0.3	8.2^a^	0.3	0.7^a^	0.4	0.5^a^	−0.4
	Serve	1.0^a^	−0.2	1.1^a^	0.2								
	Return	1.4^a^	−0.4	1.7^a^	0.4	6.2^a^	**2** **.** **6**	2.9^b^	−2.6	16.5^a^	1.0	14.7^a^	−1.0

P, pressure training; C, competition; n, number;%, percentage; CSR, corrected standard residuals; CSR > 1.96, bold.

^a,b^ Indicate significant differences in the *Z* tests for comparison of column proportions from *p* < 0.05 adjusted according to Bonferroni.

As shown in Table 4, no significant differences were observed in winners. Regarding forced errors, a higher proportion is committed in pressure training matches with the recovery smash (CSR = 2.0) and return (CSR = 2.6); whereas a higher proportion is committed in competition matches with the backhand (CSR = 2.1) and side wall backhand (CSR = 2.4). Regarding unforced errors, a higher proportion is committed in pressure training with the bandeja (CSR = 2.3).

## Discussion

The aim of the present study was to analyze the differences between pressure training and competition matches in high-level male padel players from Finland, focusing on the proportion of points won by serving and returning players, the frequency of break and golden points, and the effectiveness of the last shot.

Our findings revealed that points in both pressure training and competitive matches were most often concluded with unforced errors, followed by winners and forced errors. This distribution may be attributed to the players' proficiency level. While the prevalence of unforced errors among non-elite players has been noted previously, this study is among the first to examine the effectiveness of the last shot in high-level players who are not part of the elite cohort. In men's professional padel, previous studies have shown varied results, with winners serving as the primary point-concluding action in some tournaments (23, 29), while in others, unforced errors or a combination of forced and unforced errors dominate (24, 30). It is worth noting that earlier studies, such as Mellado Arbelo et al. (24), analyzed a limited dataset (1,060 points from 2014), and the game has likely evolved since then. Similarly, the research by Escudero-Tena et al. (30) did not differentiate between forced and unforced errors. If such a distinction had been made, winners might have emerged as the leading mechanism for concluding points, as suggested by their reported values (38.5%–44.2%, depending on the moment: non-key moment, key moment, golden point). These findings emphasize the importance of minimizing unforced errors, which remain a key performance indicator at the professional level (37).

The potential differences in the duration of pressure training and official matches may influence technical-tactical performance. While both scenarios replicate high-level gameplay, variations in match lengths could affect players' fatigue and decision-making patterns, potentially altering the comparability of performance metrics. Future studies should standardize match durations or incorporate them as covariates to better isolate performance differences between scenarios.

When serving, winners were the primary mechanism for concluding points in both match scenarios, followed by unforced and forced errors. This aligns with the tactical advantage of serving, which allows players to secure net dominance early in the rally (38). Approximately 80% of points at the professional level are won at the net (39), where serving pairs often control play and conclude points with winner shots (40). Conversely, when returning, unforced errors were the most common mechanism for point conclusion, followed by forced errors and winners. This disparity could reflect Finland's relatively recent adoption of padel and the influence of players transitioning from tennis, who may struggle to integrate wall use effectively. Furthermore, professional-level padel demonstrates a similar trend, where returning players frequently fail to dominate the net, and errors—rather than winners—tend to conclude the point (40).

Critical score situations, such as break points and golden points, play a pivotal role in padel, as they can significantly influence match outcomes (41, 42). Break points and golden points provide returning pairs with the opportunity to disrupt the server's advantage, making them high-pressure and often decisive moments. To secure a set, a pair must achieve at least one more service break than their opponents; otherwise, a tiebreak ensues at six games all, requiring at least one additional “mini-break” of serve. In this study, the frequency of break points, non-break points, golden points, and non-golden points was consistent across both pressure training and competition matches. This finding suggests that padel athletes may encounter comparable high-pressure scenarios in pressure training matches as they do in official competition.

Regarding shot types and their effectiveness, both similarities and differences were observed between pressure training and competitive matches. Smashes and recovery smashes were the primary mechanisms for winners in both contexts, highlighting their offensive importance and aligning with previous research on men's professional padel (23, 43). For forced errors, shots executed after one bounce (e.g., forehand, backhand, back wall, side wall, or double wall) were most common in both match types. However, forced errors were more frequent in pressure training with recovery smashes and returns, while competitive matches exhibited more forced errors with backhands and side wall backhands. In terms of unforced errors, volleys, bandejas, and returns were the most error-prone shots in both scenarios. Notably, unforced errors with the bandeja were more frequent during pressure training. Recent research in men's professional padel supports this finding, indicating that the bandeja often results in unforced errors at the conclusion of a point (43).

The findings of this study should be interpreted in light of the role that psychological factors, such as mental fatigue, sleep quality, and mental strength, play in shaping performance during high-pressure situations. Evidence from Díaz-García et al. and Habay et al. (44, 45) highlight the critical influence of mental fatigue on decision-making and motor performance. In addition, the impact of sleep and psychological resilience on recovery and consistency has been well documented in competitive sports (46–49). Although these aspects were not directly measured, their potential effects warrant consideration and underline the need for future research to integrate psychological assessments. Such data would complement the technical-tactical analysis, offering a more holistic understanding of player performance under competitive and training conditions.

## Strengths, limitations and future studies

This study offers several notable strengths. First, it is among the pioneering efforts to explore pressure training in the context of padel. Second, it is the first study to analyze differences between pressure training and official competition matches concerning technical-tactical parameters. Third, the practical implications of these findings are highly relevant for coaches and sport psychologists, providing valuable insights for the design of effective pressure training programs tailored to athletes' needs.

Nevertheless, certain limitations must be acknowledged. This study did not include psychological measures, such as assessments of players' mental fatigue or psychological states, which could have provided additional context for comparing pressure training and competitive matches. Additionally, not all players participated in both training and competition scenarios, which may have introduced variability. Another one is the variability in the duration of training and official matches. Although the analysis focused on a comparable dataset of points, longer or shorter matches may introduce differences in fatigue levels and strategic adjustments that were not accounted for in this study. Standardized durations in future research could enhance the validity of such comparisons. This study did not collect psychological data, such as mental fatigue, sleep quality, or mental strength, which may interact with technical-tactical performance variables. Future research should include these measures to better understand their influence on players' abilities to replicate competition scenarios during pressure training.

For future research, employing randomized controlled trials across both genders would enhance the rigor and accuracy of evaluating the impact of pressure training. Expanding the participant pool to include elite, high-level, and amateur players would also allow for a more comprehensive understanding of pressure training's effectiveness across various skill levels and demographics. Incorporating psychological assessments and exploring their interaction with technical-tactical parameters could further enrich the findings, offering a more holistic perspective on the demands of pressure training in padel.

## Conclusion

The technical-tactical performance of high-level padel players remains stable across both pressure training matches and competition matches. This stability is observed in the frequency of winners, forced errors and unforced errors, as well as the occurrence of break points, non-break points, golden points and non-golden points, regardless of whether players are serving or returning. Although minor differences in forced errors were noted, the overall shot effectiveness remained comparable. Based on these findings, it is recommended that coaches incorporate pressure training matches into their players' training regimens, as they closely replicate real competition scenarios.

## Data Availability

The raw data supporting the conclusions of this article will be made available by the authors, without undue reservation.
